# Groups of Negations on the Unit Square

**DOI:** 10.1155/2014/917432

**Published:** 2014-08-13

**Authors:** Jiachao Wu

**Affiliations:** Department of Mathematics, Shandong Normal University, Jinan 250014, China

## Abstract

The main results are about the groups of the negations on the unit square, which is considered as a bilattice. It is proven that all the automorphisms on it form a group; the set, containing the monotonic isomorphisms and the strict negations of the first (or the second or the third) kind, with the operator “composition,” is a group *G*_2_ (or *G*_3_ or *G*_4_, correspondingly). All these four kinds of mappings form a group *G*_5_. And all the groups *G*_*i*_, *i* = 2,3, 4 are normal subgroups of *G*_5_. Moreover, for *G*_5_, a generator set is given, which consists of all the involutive negations of the second kind and the standard negation of the first kind. As a subset of the unit square, the interval-valued set is also studied. Two groups are found: one group consists of all the isomorphisms on *L* ^*I*^, and the other group contains all the isomorphisms and all the strict negations on *L* ^*I*^, which keep the diagonal. Moreover, the former is a normal subgroup of the latter. And all the involutive negations on the interval-valued set form a generator set of the latter group.

## 1. Introduction

Negations, as a basic operation, play important roles in logic. In [1], the groups of the negations on the unit interval are studied. And in this paper, groups of negations on the bilattice [0,1]^2^ and on the interval-valued set *L* ^*I*^, which can be seen as a sublattice of the unit square, will be studied.

Bilattices, introduced by Ginsberg [2] as a uniform framework for inference in artificial intelligence, are algebraic structures that proved useful in many fields [3–5]. And the unit square [0,1]^2^ is a very special bilattice, which is a subset of the real plane. Thus it is of particular interest. In [6, 7], three kinds of negations on bilattices are introduced. Then one problem arises: are the properties of these negations similar to the negations on [0, 1]? In the third section of this paper, some results will be given.

All the vague sets, the interval-valued sets, the intuitionisitic fuzzy sets and the grey sets are isomorphic to sublattice *L* ^*I*^ = {(*x*_1_, *x*_2_)∈[0,1]^2^∣*x*_1_ ≤ *x*_2_}, with the natural order (*x*_1_, *x*_2_)≤_*k*_(*y*_1_, *y*_2_) if and only if *x*_1_ ≤ *y*_1_, *x*_2_ ≤ *y*_2_. Their properties could be found in [3, 7–23], and so forth. For sometimes, the sets are characterized as L* = {(*x*_1_, *x*_2_)∈[0,1]^2^∣*x*_1_ + *x*_2_ ≤ 1}, with the order (*x*_1_, *x*_2_) ≤_*t*_ (*y*_1_, *y*_2_) if and only if *x*_1_ ≤ *y*_1_, *x*_2_ ≥ *y*_2_. Both *L* ^*I*^ and L* are sublattices of the bilattice [0,1]^2^, with orders ≤_*k*_ and ≤_*t*_. Therefore, similar to [1], the groups of negations on the interval-valued sets can be discussed.

The contents will be arranged as follows. In the next section, some basic notions will be given. In the third section, the groups of the strict negations will be studied. And in the fourth section, we will study the negations on the interval-valued sets. At the end of this paper, a section of conclusion is shown.

## 2. Negations on the Bilattice [0,1]^2^

In [6, 7], three kinds of negations on the bilattice are given. As a particular bilattice, the unit square [0,1]^2^ also has these kinds of negations. These negations are based on two kinds of orders on the unit square [0,1]^2^, ≤_*t*_ and ≤_*k*_, defined as, for any *x* = (*x*_1_, *x*_2_), *y* = (*y*_1_, *y*_2_)∈[0,1]^2^,

(1) $$\begin{array}{c} \left(x_{1},x_{2}\right)\leq_{k}\left(y_{1},y_{2}\right)\;\text{if}\;x_{1}\leq y_{1},\;\;x_{2}\leq y_{2},\\ \left(x_{1},x_{2}\right)\leq_{t}\left(y_{1},y_{2}\right)\;\text{if}\;x_{1}\leq y_{1},\;\;x_{2}\geq y_{2}, \end{array}$$

in which ≤ is the natural order on [0, 1]. Actually, ≤_*k*_ is the natural order on the unit square.

**Definition 1 .**

The first kind negation *N* ^¬^, called reflection, is a unary operator on the square, satisfying the following properties: ∀*x* = (*x*_1_, *x*_2_), *y* = (*y*_1_, *y*_2_)∈[0,1]^2^,

1. if *x*≤_*k*_ *y*, then *N* ^¬^(*x*) ≥_*k*_ *N* ^¬^(*y*);
2. if *x*≤_*t*_ *y*, then *N* ^¬^(*x*) ≤_*t*_ *N* ^¬^(*y*);
3. *N* ^¬^(0,0) = (1,1) and *N* ^¬^(1,1) = (0,0).

The second kind negation *N* ^−^, named conflation, is a unary operator satisfying

(1′) if *x*≤_*k*_ *y*, then *N* ^−^(*x*)≥_*k*_ *N* ^−^(*y*);
(2′) if *x* ≤_*t*_ *y*, then *N* ^−^(*x*) ≥_*t*_ *N* ^−^(*y*);
(3′) *N* ^−^(0,0) = (1,1) and *N* ^−^(1,1) = (0,0).

The last kind negation *N* ^~^ is a unary operator satisfying

(1′′) if *x*≤_*k*_ *y*, then *N* ^~^(*x*)≤_*k*_ *N* ^~^(*y*);
(2′′) if *x* ≤_*t*_ *y*, then *N* ^~^(*x*) ≥_*t*_ *N* ^~^(*y*);
(3′′) *N* ^~^(0,0) = (0,0) and *N* ^~^(1,1) = (1,1).

For convenience, in this paper, these three kinds of negations are collectedly called negations *N* on the unit square, as a bilattice ([0,1]^2^, ≤_*k*_, ≤_*t*_).

The composition of two negations *N*, *N*′ is defined as (*N*∘*N*′)(*x*_1_, *x*_2_) = *N*(*N*′(*x*_1_, *x*_2_)). Then, it is not hard to check that *N* ^¬^∘*N* ^−^ and *N* ^−^∘*N* ^¬^ are negations of the third kind; *N* ^¬^∘*N* ^~^ and *N* ^~^∘*N* ^¬^ are the second kind negations, and *N* ^~^∘*N* ^−^ and *N* ^−^∘*N* ^~^ are of the first kind.

Similar to the definition of strict interval-valued negations in [11], the strict negations on the unit square are given as follows.

**Definition 2 .**

A negation on the unit square is called strict, if it is continuous and both ≤_*k*_ and ≤_*t*_ are strict, that is, in Definition 1, if both ≤_*k*_ and ≤_*t*_ are replaced by <_*k*_ and <_*t*_ in the premise, then the conclusion will be changed to <_*k*_ and <_*t*_ correspondingly.

**Example 3 .**

Let *N*_1_(*x*_1_, *x*_2_) = (*n*_1_(*x*_1_), *n*_2_(*x*_2_)) and *N*_2_(*x*_1_, *x*_2_) = (*n*_1_(*x*_2_), *n*_2_(*x*_1_)), with

(2) $$\begin{array}{c} n_{i}\left(a\right)=\{\begin{array}{cc} 0, & \text{if}\;a=1,\\ c_{i}, & \text{if}\;a\in \left(0,1\right),\\ 1, & \text{if}\;a=0, \end{array} \end{array}$$

in which *c*_*i*_, *i* = 1,2 are constants in [0, 1]. Then both *N*_1_ and *N*_2_ are negations, but neither of them is strict.

**Definition 4 .**

If a negation *N* satisfies *N*(*N*(*x*)) = *x*, then it is called an involutive negation.

The mapping *N*_*s*,1_ defined as *N*_*s*,1_(*x*_1_, *x*_2_) = (1 − *x*_2_, 1 − *x*_1_), for all (*x*_1_, *x*_2_)∈[0,1]^2^, is the standard negation of the first kind and named as the first standard negation. The mapping *N*_*s*,2_ defined as *N*_*s*,2_(*x*_1_, *x*_2_) = (1 − *x*_1_, 1 − *x*_2_), for all (*x*_1_, *x*_2_)∈[0,1]^2^, is called the second standard negation. And *N*_*s*,3_(*x*_1_, *x*_2_) = (*x*_2_, *x*_1_) is called the third standard kind negation. It is obvious that *N*_*s*,1_, *N*_*s*,2_, and *N*_*s*,3_ are involutive.

Obviously, each involutive negation is strict, but the converse is not valid.

**Example 5 .**

The following negation is strict but not involutive:

(3) $$\begin{array}{c} N_{3}\left(x_{1},x_{2}\right)=\left(1-x_{2}^{2},1-x_{1}\right). \end{array}$$

**Lemma 6 (see [6]).**

Each of the first kind strict negations could be characterized as *N* ^¬^(*x*_1_, *x*_2_) = (*n*_1_(*x*_2_), *n*_2_(*x*_1_)), with *n*_1_, *n*_2_ being strict negations on [0, 1], and every second kind strict negation could be characterized as *N* ^−^(*x*_1_, *x*_2_) = (*n*_1_(*x*_1_), *n*_2_(*x*_2_)), with *n*_1_ and *n*_2_ being strict negations on [0, 1].

Each of the first kind involutive negations could be characterized as *N* ^¬^(*x*_1_, *x*_2_) = (*n*_1_(*x*_2_), *n*_2_(*x*_1_)), with *n*_1_ = *n*_2_^−1^, negations on [0, 1], and every second kind involutive negation could be characterized as *N* ^−^(*x*_1_, *x*_2_) = (*n*_1_(*x*_1_), *n*_2_(*x*_2_)), with *n*_1_ and *n*_2_ being involutive negations on [0, 1].

Similarly, each of the third kind negations could be characterized as follows.

**Lemma 7 .**

*N* ^~^ is a strict negation of the third kind, if and only if there are two isomorphisms *ϕ*_1_ and *ϕ*_2_ on [0, 1], s.t.

(4) $$\begin{array}{c} N^{\sim }\left(x_{1},x_{2}\right)=\left(\phi_{1}\left(x_{2}\right),\phi_{2}\left(x_{1}\right)\right). \end{array}$$

**Proof**

Since each *N* ^~^ could be represented as *N* ^~^ = *N* ^¬^∘*N* ^−^, in which *N* ^¬^ is the first kind and *N* ^−^ is the second kind. Then from Lemma 6, the result could be obtained.

From Lemmas 6 and 7, since *n*_*i*_ and *ϕ*_*i*_, *i* = 1,2 are bijections on [0, 1], we can know each strict negation *N* is a bijection on [0,1]^2^.

Note that, the composition of two involutive negations of different kinds is still a negation, but it may not be involutive. The following example shows it.

**Example 8 .**

$N_{4}(x_{1},x_{2})=(1-x_{2}^{2},\sqrt{1-x_{1}})$ is an involutive negation of the first kind. The composition of it and the second standard negation *N*_2,*s*_,

(5) (N4∘N2,s)(x1,x2)=(1−(1−x2)2,1−(1−x1))=(2x2−x22,x1),

is a strict but not involutive negation of the third kind.

**Definition 9 .**

A continuous mapping Φ : [0,1]^2^ → [0,1]^2^ is a monotonic isomorphism on the unit square, if it is bijective and preserves both the orders ≤_*k*_ and ≤_*t*_.

Obviously, each monotonic isomorphism is continuous. Define *N*∘Φ and Φ′∘Φ as (*N*∘Φ)(*x*_1_, *x*_2_) = *N*(Φ(*x*_1_, *x*_2_)) and (Φ′∘Φ)(*x*_1_, *x*_2_) = Φ′(Φ(*x*_1_, *x*_2_)).

**Lemma 10 .**

If Φ is a monotonic isomorphism on the unit square, then there are isomorphisms *ϕ*_1_ and *ϕ*_2_ on the unit interval [0, 1] such that

(6) $$\begin{array}{c} \Phi \left(x_{1},x_{2}\right)=\left(\phi_{1}\left(x_{1}\right),\phi_{2}\left(x_{2}\right)\right). \end{array}$$

**Proof**

Firstly, let us show Φ(1,1) = (1,1), Φ(0,1) = (0,1), Φ(1,0) = (1,0), and Φ(0,0) = (0,0). For any (*x*_1_, *x*_2_) ∈ [0,1]^2^, (*x*_1_, *x*_2_)≤_*k*_(1,1). Since Φ preserves ≤_*k*_, we can know Φ(*x*_1_, *x*_2_)≤_*k*_Φ(1,1). Thus, the greatest element of (Φ([0,1]^2^), ≤_*k*_) is Φ(1,1). Because Φ is a bijection, we have Φ(1,1) = (1,1). Similarly, Φ(0,0) = (0,0), Φ(0,1) = (0,1), and Φ(1,0) = (1,0) can be proven.

Now, let us show that Φ∘*N*_*s*,1_ is a strict negation of the first kind.

Suppose (*x*_1_, *x*_2_)≤_*k*_(*y*_1_, *y*_2_) ∈ [0,1]^2^, then *N*_*s*,1_(*x*_1_, *x*_2_)≥_*k*_ *N*_*s*,1_(*y*_1_, *y*_2_). Because Φ preserves ≤_*k*_, we have

(7) (Φ∘Ns,1)(x1,x2)=Φ(Ns,1(x1,x2))≥kΦ(Ns,1(y1,y2))=(Φ∘Ns,1)(y1,y2).

It shows that Φ∘*N*_*s*,1_ reverses the order ≤_*k*_. Similarly, since both Φ and *N*_*s*,1_ keep the order ≤_*t*_, we can get that Φ∘*N*_*s*,1_ preserves the order ≤_*t*_. Since Φ is a bijection, Φ strictly preserves the orders ≤_*k*_ and ≤_*t*_. Similarly, it can be proven that Φ∘*N*_*s*,1_ strictly preserves the order ≤_*t*_ and strictly reverses the order ≤_*k*_.

Since both Φ and *N*_*s*,1_ are continuous, Φ∘*N*_*s*,1_ is continuous.

Since Φ(1,1) = (1,1), Φ(0,0) = (0,0), and *N*_*s*,1_(1,1) = (0,0), *N*_*s*,1_(0,0) = (1,1), the following hold:

(8) $$\begin{array}{c} \left(\Phi \circ N_{s,1}\right)\left(0,0\right)=\Phi \left(N_{s,1}\left(0,0\right)\right)=\left(1,1\right),\\ \left(\Phi \circ N_{s,1}\right)\left(1,1\right)=\Phi \left(N_{s,1}\left(1,1\right)\right)=\left(0,0\right). \end{array}$$

These two formulas, together with the facts that Φ∘*N*_*s*,1_ is continuous, strictly reverses the order ≤_*k*_, and strictly preserves the order ≤_*t*_, show that Φ∘*N*_*s*,1_ is a strict negation of the first kind, denoted by *N* ^¬^ = Φ∘*N*_*s*,1_.

Then for any (*x*_1_, *x*_2_) ∈ [0,1]^2^, (*N* ^¬^∘*N*_*s*,1_)(*x*_1_, *x*_2_) = ([Φ∘*N*_*s*,1_]∘*N*_*s*,1_)(*x*_1_, *x*_2_) = Φ(*N*_*s*,1_(*N*_*s*,1_(*x*_1_, *x*_2_))) = Φ(*x*_1_, *x*_2_), that is, Φ = *N* ^¬^∘*N*_*s*,1_. From Lemma 6, *N* ^¬^(*x*_1_, *x*_2_) = (*n*_1_(*x*_2_), *n*_2_(*x*_1_)), with *n*_1_, *n*_2_ being negations on the unit interval [0, 1]. Since *N*_*s*,1_(*x*_1_, *x*_2_) = (1 − *x*_2_, 1 − *x*_1_), we could get Φ(*x*_1_, *x*_2_) = (*n*_1_(1 − *x*_1_), *n*_2_(1 − *x*_2_)). Because *n*_1_(1 − *x*_1_) and *n*_2_(1 − *x*_2_) are isomorphisms on the unit interval, Φ can be characterized as Φ(*x*_1_, *x*_2_) = (*ϕ*_1_(*x*_1_),  *ϕ*_2_(*x*_2_)), in which *ϕ*_1_(*x*_1_) = *n*_1_(1 − *x*_1_), *ϕ*_2_(*x*_2_) = *n*_2_(1 − *x*_2_) are isomorphisms on the unit interval.

**Lemma 11 .**

If Φ is a monotonic isomorphism and *N*_*s*_ is a standard negation, then Φ∘*N*_*s*_ and *N*_*s*_∘Φ are strict negations and of the same kind as *N*_*s*_. Conversely, for each strict negation *N*, there exists a monotonic isomorphism Φ and a standard negation *N*_*s*_ of the same kind with *N*, s.t. *N* = Φ∘*N*_*s*_.

**Proof**

If *N*_*s*_ = *N*_*s*,1_, the proof of Lemma 10 has already shown that Φ∘*N*_*s*,1_ is a strict negation. And by Lemma 10, (*N*_*s*,1_∘Φ)(*x*_1_, *x*_2_) = (1 − *ϕ*(*x*_2_), 1 − *ϕ*(*x*_1_)). It shows that *N*_*s*,1_∘Φ is a continuous bijection on [0,1]^2^, which strictly preserves the order ≤_*k*_ and strictly reverses ≤_*t*_ and satisfies (*N*_*s*,1_∘Φ)(1,1) = (0,0) and (*N*_*s*,1_∘Φ)(0,0) = (1,1). Therefore, *N*_*s*,1_∘Φ is a strict negation of the first kind.

Similar for *N*_*s*_ = *N*_*s*,2_ or *N*_*s*_ = *N*_*s*,3_. Therefore, Φ∘*N*_*s*_ and *N*_*s*_∘Φ are strict negations and of the same kind as *N*_*s*_, for *N*_*s*_ = *N*_*s*,*i*_,  *i* = 1,  2,  3.

Next, let us show the second part. Suppose *N* is a negation and *N*_*s*_ is the standard negation of the same kind as *N*. Then, *N*∘*N*_*s*_ is a monotonic isomorphism Φ. As a result, for any (*x*_1_, *x*_2_) ∈ [0,1]^2^,

(9) (Φ∘Ns)(x1,x2)=([N∘Ns]∘Ns)(x1,x2)=N[Ns(Ns(x1,x2))]=N(x1,x2),

that is, *N* = Φ∘*N*_*s*_.

## 3. Groups of the Negations on the Unit Square

Based on the notions in the above section, the groups of the negations on the unit square will be discussed.

In [1], the following theorem is obtained.

**Theorem 12 (see [1, Theorem 4]).**

(*1*) For every strict negation *n* on the unit interval, there exist three involutive negations *n*_1_, *n*_2_ and *n*_3_ s.t. *n* = *n*_1_∘*n*_2_∘*n*_3_.

(*2*) For every isomophism *ϕ* on the unit interval, there exist four involutive negations *n*_1_, *n*_2_, *n*_3_ and *n*_4_ s.t. *ϕ* = *n*_1_∘*n*_2_∘*n*_3_∘*n*_4_.

This theorem shows that under the operator “composition,” all the involutive negations and isomorphisms on the unit interval cannot form a group, since it is not closed under the operator “composition.” But the set of all the strict negations and isomorphisms, together with the operator “composition,” is a group [1].

From this theorem and Lemmas 6 and 10, we could get the following result on the unit square.

**Theorem 13 .**

(*1*) For every strict negation *N* of the second kind on the unit square, there exist three involutive negations *N*_1_, *N*_2_, and *N*_3_ of the second kind, s.t. *N* = *N*_1_∘*N*_2_∘*N*_3_.

(*2*) For every monotonic isomorphism Φ on the unit square, there exist four involutive negations *N*_1_, *N*_2_, *N*_3_, and *N*_4_ of the second, s.t. Φ = *N*_1_∘*N*_2_∘*N*_3_∘*N*_4_.

For the other two kinds of negations, we have not got similar results, since the composition operator is not commutative.

Next, let us discuss the groups of the negations on the unit square. The following sets are denoted by

 
* S*_1_ = {Φ : Φ is a monotonic isomorphism on the unit square.}
 
* S*_2_ = *S*_1_ ∪ {*N* ^¬^ : *N* ^¬^ is a first kind strict negation on the unit square.}
 
* S*_3_ = *S*_1_ ∪ {*N* ^−^ : *N* ^−^ is a second kind strict negation on the unit square.}
 
* S*_4_ = *S*_1_ ∪ {*N* ^~^ : *N* ^~^ is a third kind strict negation on the unit square.}
 
* S*_5_ = *S*_2_ ∪ *S*_3_ ∪ *S*_4_.

**Theorem 14 .**

*G*_*i*_ = (*S*_*i*_, ∘), *i* = 1,2, 3,4, 5 are groups, with the same unit element id. Moreover, *G*_1_◃*G*_2,3,4,5_◃*G*_5_, that is, *G*_1_ is a normal subgroup of *G*_*i*_, *i* = 2,3, 4,5, and *G*_2,3,4,5_ are normal subgroups of *G*_5_.

**Proof**

Obviously, for any element *f* in *G*_*i*_, *i* = 1,…, 5, id∘*f* = *f*∘id = *f*, that is, id is the unit element of *G*_*i*_, *i* = 1,…, 5.

For any two monotonic isomorphisms Φ_1_, Φ_2_, the composition of them is still a monotonic isomorphism. Thus *G*_1_ is closed under the operator “∘”. For two strict negations *N*_1_, *N*_2_ of the same kind, the composition of them is also a monotonic isomorphism. The composition of a strict negation *N* and a monotonic isomorphism Φ is still a strict negation of the same kind with *N*. Therefore, *G*_2_, *G*_3_, and *G*_4_ are closed under the operator “∘”. For any two strict negations *N*_1_, *N*_2_ of different kind, the composition is a strict negation of the other kind. So *G*_5_ is closed.

Now, let us prove the associativity of *G*_5_. This proof also shows the associativity of *G*_*i*_, *i* = 1,2, 3,4.

From Lemmas 6, 7, and 10, each of the negations and the monotonic isomorphisms can be characterized as *F*(*x*_1_, *x*_2_) = (*f*_1_(*x*_1_), *f*_2_(*x*_2_)) or *F*(*x*_1_, *x*_2_) = (*f*_1_(*x*_2_), *f*_2_(*x*_1_)), in which both of *f*_1_ and *f*_2_ are isomorphisms on [0, 1] or both are strict negations on [0, 1]. Let *F*_1_, *F*_2_, *F*_3_ be negations or monotonic isomorphisms on [0,1]^2^. Then,

(10) (F1∘[F2∘F3])(x1,x2)=F1[(F2∘F3)(x1,x2)]=F1[F2(F3(x1,x2))]=[F1∘F2](F3(x1,x2))=([F1∘F2]∘F3)(x1,x2),

that is, *F*_1_∘[*F*_2_∘*F*_3_] = [*F*_1_∘*F*_2_]∘*F*_3_, which shows the associativity of *G*_5_.

For any monotonic isomorphism Φ and any strict negation *N*, obviously Φ^−1^ and *N* ^−1^ exist. Moreover, Φ^−1^ is also a monotonic isomorphism and *N* ^−1^ is a negation of the same kind as *N*, that is, the inverse about the composition operator “∘” exists. Therefore, *G*_*i*_, *i* = 1,…, 5 are groups.

Since *S*_1_ ⊂ *S*_2,3,4_ ⊂ *S*_5_, *G*_1_ < *G*_2,3,4_ < *G*_5_, we only need to prove that they are normal subgroups.

Firstly, let us show *G*_1_◃*G*_2_. For any Φ ∈ *S*_1_, obviously, Φ∘*G*_1_∘Φ^−1^ ⊂ *G*_1_, because *G*_1_ is a group. For *N* ∈ *S*_2_∖*S*_1_, from Lemma 6, we know *N*(*x*_1_, *x*_2_) = (*n*_1_(*x*_2_), *n*_2_(*x*_1_)) and *N* ^−1^(*x*_1_, *x*_2_) = (*n*_1_^−1^(*x*_2_), *n*_2_^−1^(*x*_1_)). For any Φ(*x*_1_, *x*_2_) = (*ϕ*_1_(*x*_1_), *ϕ*_2_(*x*_2_)) ∈ *S*_1_,

(11) (N∘Φ∘N−1)(x1,x2)  =(n1(ϕ2(n2−1(x1))),n2(ϕ1(n1−1(x2)))).

Because both *n*_1_∘*ϕ*_2_∘*n*_2_^−1^ and *n*_2_∘*ϕ*_1_∘*n*_1_^−1^ are isomorphisms on the unit interval [0, 1], *N*∘Φ∘*N* ^−1^ is in *S*_1_. Thus, *N*∘*G*_1_∘*N* ^−1^ ⊂ *G*_1_, that is, *G*_1_ is a normal subgroup of *G*_2_.

Similarly, we could prove *G*_1_◃*G*_3,4_. Since, *S*_5_ = *S*_2_ ∪ *S*_3_ ∪ *S*_4_, we could know *G*_1_◃*G*_5_.

Now, let us show *G*_2_◃*G*_5_. Because *G*_2_ is a group, for all *f* ∈ *G*_2_ (*f* is a strict negation of the first kind or a monotonic isomorphism on [0,1]^2^), we have *f*∘*G*_2_∘*f* ^−1^ ⊂ *G*_2_. For any *N* ∈ *G*_5_∖*G*_2_, *N* is a negation of the second kind or the third kind. If *N* is of the second kind, then *N* ^−1^ is also of the second kind. Thus for all Φ ∈ *G*_2_, *N*∘Φ∘*N* ^−1^ ∈ *G*_2_, and for all *N*′ ∈ *G*_2_, *N*∘*N*′∘*N* ^−1^ is of the first kind, since *N*′∘*N* ^−1^ is of the third kind. If *N* is of the third kind, then *N* ^−1^ is also of the third kind. Thus for all Φ ∈ *G*_2_, *N*∘Φ∘*N* ^−1^ ∈ *G*_2_, and for all *N*′ ∈ *G*_2_, *N*∘*N*′∘*N* ^−1^ is of the first kind, since *N*′∘*N* ^−1^ is of the second kind. Therefore, for all *f* ∈ *G*_5_, *f*∘*G*_2_∘*f* ^−1^ ⊂ *G*_2_, that is, *G*_2_ is a normal subgroup of *G*_5_. Similarly, *G*_3_◃*G*_5_, *G*_4_◃*G*_5_ could be proven.

From Theorem 13, we immediately get the following result.

**Theorem 15 .**

The set *S*_*I*,2_ of all the involutive negations of the second kind could generate the group *G*_3_, that is, for any *f* ∈ *G*_3_, there is some involutive negations *N*_*i*_, *i* = 1,…, *m* of the second kind, such that *f* = *N*_1_∘*N*_2_∘⋯∘*N*_*m*_. And if *f* is a negation, *m* could be 3; if *f* is an isomorphism, *m* could be 4.

This theorem shows the relation between the involutive negations of the second kind and the group *G*_3_. However, the following problem is still unproven.

**Problem 16 .**

Could *G*_2_ (or *G*_4_) be generated by the set of all the involutive negations of the first (or the third) kind?

A generator set of *G*_5_ is given in the following theorem.

**Theorem 17 .**

The set *S*_*I*,2_ ∪ {*N*_*s*,1_} is a generator set of the group *G*_5_. And *S*_*I*,2_ ∪ {*N*_*s*,3_} is also a the generator set of *G*_5_.

**Proof**

By Lemma 11, for any strict negation *N* of the first or the third kind, there exists a monotonic isomorphism Φ, s.t. *N* = *N*_*s*,1_∘Φ or *N* = *N*_*s*,2_∘Φ. Since *N*_*s*,1_∘*N*_*s*,2_ = *N*_*s*,3_, from Theorem 15, *S*_*I*,2_ ∪ {*N*_*s*,1_} generates the group *G*_5_.

Because *N*_*s*,2_∘*N*_*s*,3_ = *N*_*s*,1_, *S*_*I*,2_ ∪ {*N*_*s*,3_} is also a the generator set of *G*_5_.

## 4. Negations on the Interval-Valued Set

The interval-valued set *L* ^*I*^ is a sublattice of the unit square [0,1]^2^, so the negations of the interval-valued set could be defined as the restriction of the negations on the unit square.

**Definition 18 .**

A negation *N* ^*I*^ on the interval-valued set is the restriction of some negation *N* of the first kind on the unit square, and satisfies that

(12) $$\begin{array}{c} \forall x\in L^{I},\;N^{I}\left(x\right)={N|}_{L^{I}}\left(x\right)\in L^{I}. \end{array}$$

**Definition 19 .**

An interval-valued negation is strict, if it is the restriction of some strict negations of the first kind on the unit square and satisfies (12).

An interval-valued negation *N* ^*I*^ is involutive, if it satisfies

(13) $$\begin{array}{c} N^{I}\left(N^{I}\left(x\right)\right)=x,\;\forall x\in L^{I}. \end{array}$$

From this definition, each strict interval-valued negation is an injection and each involutive negation is strict [6]. Also, in [6], it is proven that for each involutive interval-valued negation *N* ^*I*^, it keeps the diagonal Δ = {(*x*_1_, *x*_2_) ∈ *L* ^*I*^ : *x*_1_ = *x*_2_}, that is,

(14) $$\begin{array}{c} \forall x\in \Delta ,\;N^{I}\left(x\right)\in \Delta . \end{array}$$

**Definition 20 .**

A mapping Φ^*I*^ on the interval-valued set is an isomorphism, if it is a bijection and keeps the natural order.

Actually, if Φ^*I*^ is an isomorphism on the interval-valued set, then Φ^*I*^ keeps both the orders ≤_*k*_ and ≤_*t*_ [6]. Thus, there exist some isomorphisms Φ on the unit square, s.t. Φ^*I*^ = Φ|_*L*^*I*^_. Moreover, every Φ keeps the diagonal Δ [6].

Different from the unit square, all the strict negations and the isomorphisms on the interval-valued set, together with the composition operator, do not consist of a group. The following is a counter example.

**Example 21 .**

There is no strict negation on the interval-valued set, which is an inverse of the following negation *N*_1_^*I*^:

(15) $$\begin{array}{c} N_{1}^{I}\left(x_{1},x_{2}\right)=\left(1-x_{2},1-x_{1}^{2}\right),\;\forall \left(x_{1},x_{2}\right)\in L^{I}. \end{array}$$

It is not hard to check that *N*_1_^*I*^ is a strict negation on the interval-valued set.

It seems that there is a mapping $F_{1}(x_{1},x_{2})=(\sqrt{1-x_{2}^{2}},1-x_{1})$, s.t. *F*_1_ is its inverse. However, not all of the points of the interval-valued set are well defined under *F*_1_, such as the point (0.5, 0.5). The “image” of it is (0.75, 0.5), which is out of the interval-valued set.

Also, the following mapping *F*_2_ is also not the inverse of *N*_1_^*I*^, because it is not an injection, thus not a strict negation on *L* ^*I*^. Consider

(16) $$\begin{array}{c} F_{2}\left(x_{1},x_{2}\right)=\{\begin{array}{cc} \left(\sqrt{1-x_{2}^{2}},1-x_{1}\right), & \text{if}\;{\left(1-x_{1}\right)}^{2}\geq 1-x_{2},\\ \left(x_{1},x_{1}\right), & \text{otherwise}. \end{array} \end{array}$$

Now, let us give the proof of Example 21.

Suppose *N*_2_^*I*^ is the inverse negation of *N*_1_^*I*^. Clearly, *N*_1_^*I*^ maps the interval-valued set *L* ^*I*^ to the set *S* = {(*x*_1_, *x*_2_) : 0 ≤ 1 − *x*_2_ ≤ (1−*x*_1_)^2^ ≤ 1}. Then (*N*_2_^*I*^)|_*S*_ is a surjection *S* to *L* ^*I*^. Since for the points in *L* ^*I*^∖*S*, their images under *N*_2_^*I*^ also should be in *L* ^*I*^, we can get that *N*_2_^*I*^ is not an injection, thus not a strict negation on *L* ^*I*^. So *N*_1_^*I*^ has no inverse.

Denote that

 
* S*_1_^*I*^ = {Φ^*I*^ : Φ  is  an  isomorphism  *on*⁡  *L* ^*I*^.}
 
* S*_2_^*I*^ = *S*_1_^*I*^ ∪ {*N* ^*I*^ : *N* ^*I*^ is a strict negation on *L* ^*I*^ and keeps the diagonal Δ.}

Then we have the following theorem.

**Theorem 22 .**

*G*_1_^*I*^ = (*S*_1_^*I*^, ∘) and *G*_2_^*I*^ = (*S*_2_^*I*^, ∘) are groups, with ∘ the composition of the mappings. Moreover, *G*_1_^*I*^◃*G*_2_^*I*^.

**Proof**

Obviously, the unit element is the identity mapping id, and the operation is closed and associative.

Suppose Φ^*I*^ is an isomorphism in *S*_1_^*I*^. Then Φ^*I*^ can be represented as Φ^*I*^(*x*_1_, *x*_2_) = (*ϕ*(*x*_1_), *ϕ*(*x*_2_)), with *ϕ* an isomorphism on the unit interval [0, 1], because Φ^*I*^ keeps the diagonal. Define the mapping (Φ^*I*^)^−1^ as (Φ^*I*^)^−1^(*x*_1_, *x*_2_) = (*ϕ* ^−1^(*x*_1_), *ϕ* ^−1^(*x*_2_)). Then (Φ^*I*^)^−1^ is also an isomorphism on *L* ^*I*^, that is, (Φ^*I*^)^−1^ ∈ *S* ^*I*^, and

(17) $$\begin{array}{c} \left[{\left(\Phi^{I}\right)}^{-1}\circ \Phi^{I}\right]\left(x_{1},x_{2}\right)=\left(x_{1},x_{2}\right)=\left[\Phi^{I}\circ {\left(\Phi^{I}\right)}^{-1}\right]\left(x_{1},x_{2}\right), \end{array}$$

that is, (Φ^*I*^)^−1^ is the inverse of Φ^*I*^. Thus, *G*_1_^*I*^ is a group.

Let *N* ^*I*^ be a strict negation on *L* ^*I*^, which keeps the diagonal Δ. From Lemma 6, there exist some strict negations *n*_1_, *n*_2_ on [0, 1], s.t. *N* ^*I*^(*x*_1_, *x*_2_) = (*n*_1_(*x*_2_), *n*_2_(*x*_1_)). From (14), *n*_1_ = *n*_2_. Then (*N* ^*I*^)^−1^(*x*_1_, *x*_2_) = (*n*_1_^−1^(*x*_2_), *n*_1_^−1^(*x*_1_)) is also a strict negation on *L* ^*I*^ and keeps the diagonal. Also we can check that

(18) $$\begin{array}{c} \left[{\left(N^{I}\right)}^{-1}\circ N^{I}\right]\left(x_{1},x_{2}\right)=\left(x_{1},x_{2}\right)=\left[N^{I}\circ {\left(N^{I}\right)}^{-1}\right]\left(x_{1},x_{2}\right); \end{array}$$

that is, (*N* ^*I*^)^−1^ is the inverse of *N* ^*I*^. Thus, *G*_2_^*I*^ is a group.

Similar to the proof of Theorem 14, *G*_1_^*I*^◃*G*_2_^*I*^ could be proven.

This theorem could be extended to the unit square.

**Theorem 23 .**

(*1*) All the monotonic isomorphisms on the unit square, which keep the diagonal, form a group, called *G*_6_.

(*2*) All the strict negations of the first kind and the monotonic isomorphisms on the unit square, which keep the diagonal, form a group, called *G*_7_.

The proof is similar to Theorem 22.

From Theorems 12, 22, and 23, we could obtain the following theorem.

**Theorem 24 .**

(*1*) The set of all the involutive negations on *L* ^*I*^ generates the group *G*_2_^*I*^.

(*2*) The set of all the involutive negations of the first kind on the unit square, which keep the diagonal, generates the group *G*_7_.

## 5. Conclusion

In this paper, we firstly study the negations on the unit square. The main results are Theorems 14 and 23, which show the groups that are formed by the strict negations and the monotonic isomorphisms. Then we discuss the negations on the interval-valued set. The main result is Theorem 22, that is, all the strict negations and isomorphisms on *L* ^*I*^, which keep the diagonal, form a group. Moreover, some generator sets of the groups are given.
